# Nanoparticle biofabrication using English ivy (*Hedera helix*)

**DOI:** 10.1186/1477-3155-10-41

**Published:** 2012-10-24

**Authors:** Jason N Burris, Scott C Lenaghan, Mingjun Zhang, C Neal Stewart

**Affiliations:** 1 Department of Plant Sciences, University of Tennessee, 252 Ellington Plant Sciences, 2431 Joe Johnson Drive, Knoxville, TN, 37996, USA; 2 Department of Mechanical, Aerospace and Biomedical Engineering, University of Tennessee, Knoxville, TN, 37996, USA

## Abstract

**Background:**

English ivy (*Hedera helix*) is well known for its adhesive properties and climbing ability. Essential to its ability to adhere to vertical surfaces is the secretion of a nanocomposite adhesive containing spherical nanoparticles, 60–85 nm in diameter, produced exclusively by root hairs present on adventitious roots. These organic nanoparticles have shown promise in biomedical and cosmetic applications, and represent a safer alternative to metal oxide nanoparticles currently available.

**Results:**

It was discovered that the maximum adventitious root production was achieved by a 4 h application of 1 mg/ml indole-3 butyric acid (IBA) to juvenile English ivy shoot segments cultured in custom vessels. After incubation of the shoots under continuous light at 83 μmol/m^2^ s at 20°C for 2 weeks, the adventitious roots were harvested from the culture system and it was possible to isolate 90 mg of dry weight nanoparticles per 12 g of roots. The nanoparticle morphology was characterized by atomic force microscopy, and found to be similar to previous studies.

**Conclusions:**

An enhanced system for the production of English ivy adventitious roots and their nanoparticles by modifying GA7 Magenta boxes and identifying the optimal concentration of IBA for adventitious root growth was developed. This system is the first such platform for growing and harvesting organic nanoparticles from plants, and represents an important step in the development of plant-based nanomanufacturing. It is a significant improvement on the exploitation of plant systems for the formation of metallic nanoparticles, and represents a pathway for the generation of bulk ivy nanoparticles for translation into biomedical applications.

## Background

A wide variety of plants across several taxa have been shown to produce metal nanoparticles with interesting properties when combined with silver nitrate or gold (III) chloride. Nanoparticle production, without the need for silver or gold, has been demonstrated in sundew [1] and English ivy (*Hedera helix* L.; family, Araliaceae) [2-5], a climbing plant well known for its ability to adhere to vertical surfaces [6]. Recent research has demonstrated that the adventitious roots of English ivy are responsible for the production of an adhesive compound composed of polysaccharide and spherical nanoparticles 60–85 nm in diameter [4,5]. These organic nanoparticles have an optical absorption and light scattering properties that make them attractive candidates for sunscreen fillers, especially in light of the toxicity concerns over currently available TiO_2_ and ZnO nanoparticles [7,8]. In addition to sunscreen applications, the strong adhesive properties of the nanocomposite adhesive formed from the English ivy nanoparticles and surrounding polysaccharide matrix have been implicated in both biomedical and traditional adhesive applications. In both cases, natural nanoparticles produced in plants might be attractive alternatives to currently-used metal nanoparticles [7,8].

Prior to this study it has been laborious to obtain sufficient homogeneous nanoparticles for research purposes. Therefore, our goal was to develop an effective system for nanoparticle production using English ivy as a bioproduction factory. In this work, special attention has been paid to the effect of exogenous auxin application, in this case as a stem soak of indole-3 butyric acid (IBA), to optimize adventitious root and nanoparticle production. Further, a growth-culture system for nanoparticle production using modified Magenta GA7 tissue culture vessels was developed.

## Results and discussion

**Figure 1:**
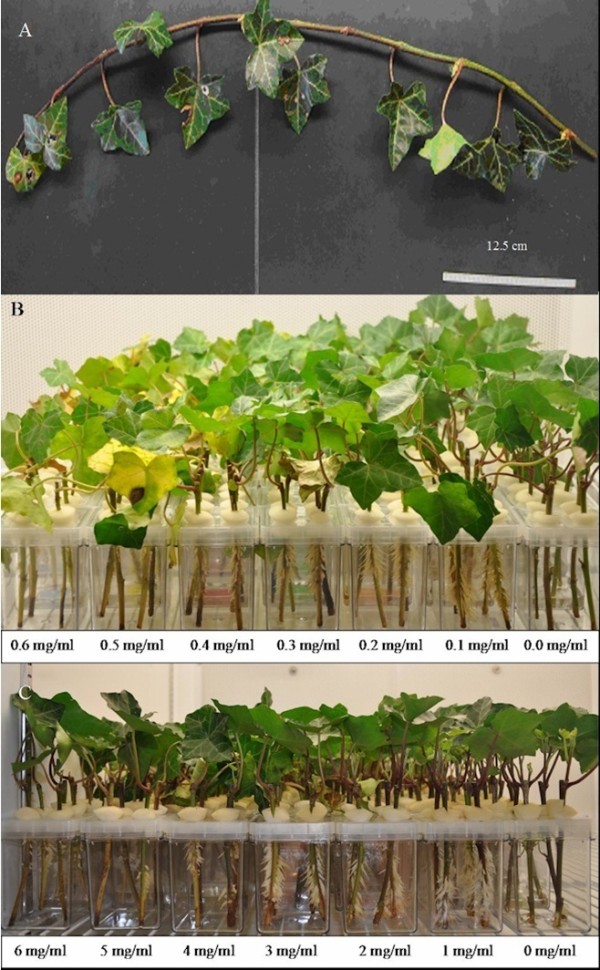
***H*. *helix *adventitious root production after 2 weeks.A.** Initial 50 cm stems were cut into 12.5 cm pieces and treated with either **B.** low levels of IBA overnight or **C.** high levels of IBA for 4 h.

**Figure 2:**
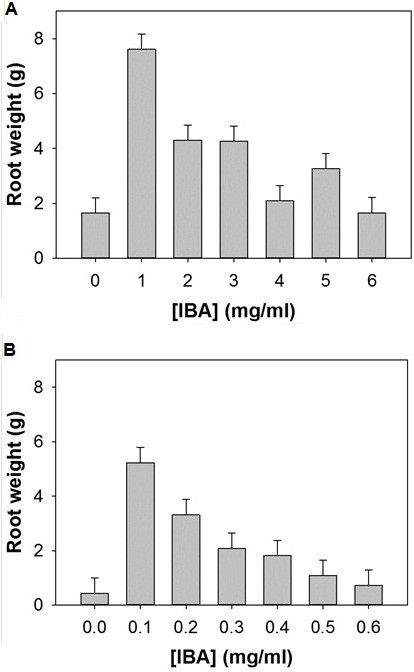
***H*. *helix *adventitious root production by weight (g) treated with either A. high levels of IBA ([0–6 mg/ml]) for 4 h or B. low levels of IBA ([0–0.6 mg/ml]) for 16 h.** Error bars represent 95% confidence intervals using least significant differences (P<0.05).

**Figure 3:**
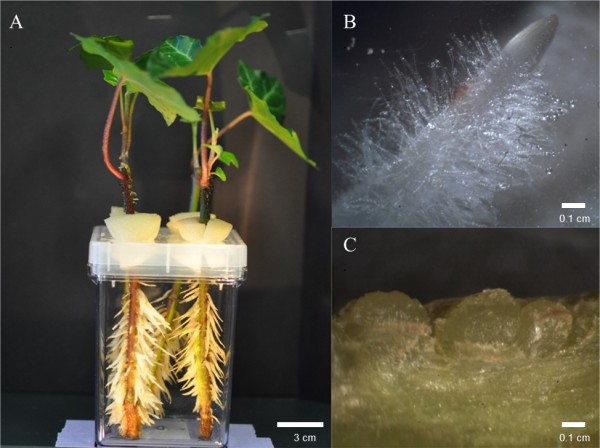
**Adventitious roots produced from *H*. *helix *A. after 2 weeks treated with 100 mg IBA for 4 h, B. adventitious roots releasing adhesive, and C. root primordial after 1 week**.

**Figure 4:**
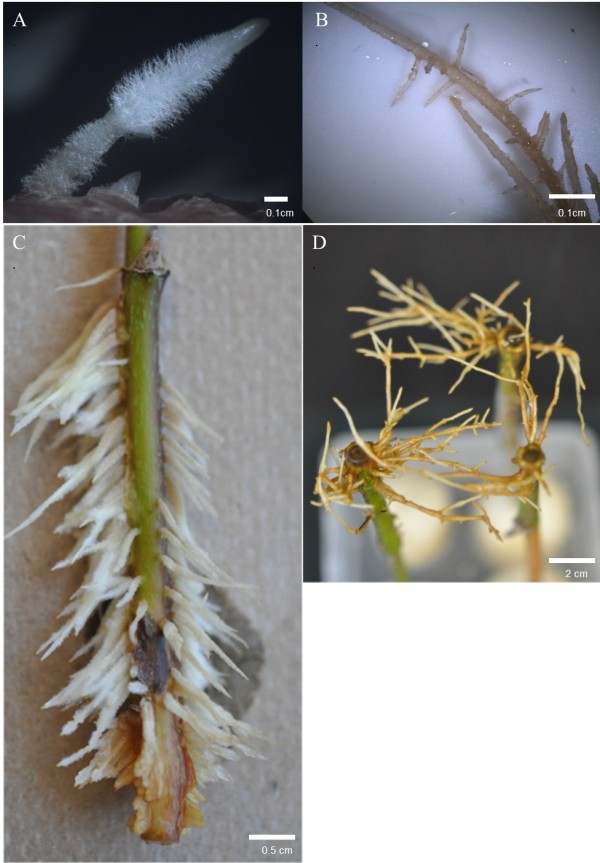
**Two root types produced by *H*. helix.A.** adventitious and **B.** subterranean roots as viewed under a light microscope. **C.** High levels of IBA shoots and **D.** subterranean roots.

Figure 1 shows *Hedera helix* adventitious root production after an incubation of 2 weeks from shoots cut into 12.5 cm segments and treated with either low concentrations of IBA 0.0, 0.1, 0.2, 0.3, 0.4, 0.5, 0.6 mg/ml for 16 h or high concentrations of IBA 0, 10, 20, 30, 40, 50, 60, mg/ml after 4 h. Delaying the processing of stems beyond the day received was shown to affect the production of adventitious roots (data not shown), likely an abiotic stress response. Maximum adventitious root production (frozen weight) was achieved by soaking juvenile stems in IBA at a concentration of 1 mg/ml for 4 h, producing approximately 8 g adventitious roots per 5 GA7 vessels (Figure 1 and 2), over 100 roots per stem (data not shown) when tallied. Adventitious root production was optimal under a relatively high IBA concentration of 1 mg/ml for 4 h periods (Figure 2). Adventitious roots produced using the 1 mg/ml soak over 4 h led to normal adhesive material release with adhesion to the GA7 vessel wall (Figure 3). Similar to observations by previous researchers [5], no root hairs were observed to be produced at the tips of roots (Figures 3B, 4A).

**Figure 5:**
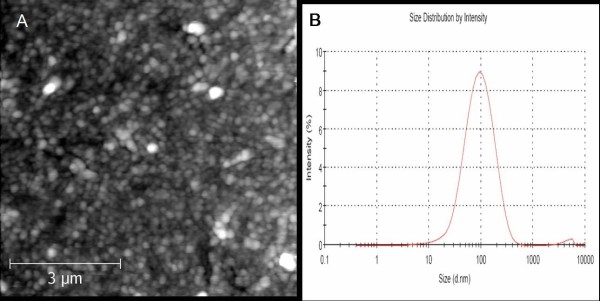
**AFM and DLS of isolated ivy nanoparticles.A.** AFM micrograph of ivy nanoparticles. **B.** DLS of ivy nanoparticles, with a mean diameter of 109.8 ± 5.6 nm.

Two types of roots are produced from *H*. *helix*—adventitious and subterranean (Figure 4). Subterranean roots lack root hairs and produce a branching pattern not observed in adventitious roots (Figure 4). Previous research, employing a real-time observation system, demonstrated nanoparticles are released specifically from adventitious root hairs [5]. Therefore, we created a system designed for the enhanced production of adventitious roots and associated root hairs. Researchers have hypothesized that English ivy attachment to vertical surfaces occurs in four stages [9], and recent research [5] determined the secretion process took approximately 4 to 6 h, and observed that adhesive droplets from multiple root hairs in close proximity fused to form larger adhesive droplets. Similarly, we observed the formation of adhesive droplets on the adventitious roots. The morphology of English ivy nanoparticles were analyzed by atomic force microscopy and dynamic light scattering (Figure 5) and showed similar results to what has been previous shown from both natural and tissue culture produced roots [1-5].

In natural conditions in the absence of root-to-surface contact, adventitious roots can grow unbranched to lengths 1–15 mm [9]. We have observed root growth of greater than 30 mm without the release of nanoparticles in the GA7 boxes, because of the artificial conditions placed on the stems and excess humidity provided by our rooting chambers. Adventitious rooting cuttings are normally placed under intermittent misting systems that spray water for 2.5 sec every 5 min to ensure humidity is kept high [10]. By omitting the need for misting and soil/media substrate we have created ideal conditions where cultivated roots produce intact nanoparticles until harvest or until application of mechanical stimulus for natural release. In our observations of roots produced on potted plants, roots that do not come in contact with an attachable surface will dehydrate and abort. Therefore, a high humidity system is required for optimal adventitious root and nanoparticle production. Under high humidity, roots grow unabated for at least one month.

While the composition of the nanoparticle and polysaccharide components in English ivy are unknown, Virginia creeper (*Parthenocissus quinquefolia* L.; family, Vitaceae) exudes a debranched rhamnogalacturonan (RG) I, which allows its attachment to vertical surfaces [11,12]. In order to characterize the chemical composition of English ivy nanoparticles and secreted polysaccharides, it is necessary to produce sufficient quantities of secreted materials for chemical and physical analysis. As such, it was necessary to develop a production and purification system as a means of producing large quantities of adventitious roots and nanoparticles. Here we designed and manufactured a simple rooting chamber for English ivy adventitious root production. In the past, *H*. *helix* has been examined for its adventitious rooting properties for the production of cuttings for the ornamental horticulture industry [13,14], whereby adventitious roots were produced at very low concentrations, 12 to 22 roots per stem, based upon the treatment applied [10,14]. Prior research examined the cuttings for production in a horticultural setting through the use of a potting media with and without a misting system [10]. In this study, the addition of IBA and the development of ivy rooting chambers was a significant advancement allowing for the production of large quantities of adventitious roots, and thus ivy nanoparticles.

## Methods

Initial English ivy propagules were provided by David Gilmore (Swan Valley Farms, Mount Vernon, Washington, USA). Whole above ground portions of plants were harvested in Washington, placed into shipment boxes and shipped overnight to Knoxville, Tennessee. Upon arriving the next day, plants were unpacked and stems were segmented to 12.5 cm linear sections and leaves were removed except for one leaf at each stem’s apex. IBA potassium salt (Sigma, St. Louis, Missouri, USA) stock solutions were prepared at 50 mg/ml. Two types of IBA soak procedures were performed: an overnight 16 h soak at a low concentration and a 4 h soak with a high concentration. For each concentration, the appropriate amount of a stock solution of 50 mg/ml IBA was added to deionized water for a final volume of 100 ml. For the low concentration soak, concentrations of 0, 0.1, 0.2, 0.3, 0.4, 0.5 and 0.6 mg/ml of IBA were used. For the high concentration soak, concentrations of 0, 1, 2, 3, 4, 5, and 6 mg/ml of IBA were used. Stems were placed in 150 ml beakers so that the solution covered 75% of the stems (apex was dry) and were incubated in a darkened room overnight. Post-treatment, four stem segments were placed per Magenta GA7 box (Fisher, Waltham, Massachusetts, USA) in an incubator for continuous light at 83 μmol/m^2^ s at 20°C for 2 weeks. Magenta boxes were converted to ivy rooting chambers by drilling lids with four 13 mm holes. Each hole was centered 1.5 mm from respective corners and a foam plug that has been cut to the center was placed into each hole. Stems were placed into the foam plugs and the bottom of stems rested on the bottom of the Magenta box. Fifty milliliters of water were placed in each box to provide humidity and moisture.

Following 2 weeks growth, roots were harvested from stems, flash frozen in liquid nitrogen and fresh weights were recorded. Nanoparticles were extracted from 12 g of frozen adventitious rootlets and macerated as described in [8]. Macerated tissue was squeezed using a glass dounce tissue grinder, only the liquid from the tissue was collected (approximately 10 ml), centrifuged at 5000 x g for 10 min and filtered through a 0.22 μm filter. Nanoparticles were then dialyzed in Spectra cellulose ester dialysis membranes MWCO 300,000 against DI water overnight with 3 DI water changes. Solutions were frozen at −80C, lyophilized (FreeZone 12 L, Labconco) and dry weights were recorded. The presence and morphology of ivy nanoparticles produced using the system described above were analyzed by atomic force microscopy and dynamic light scattering, as defined in [7,8].

Data were analyzed as a completely randomized design with two replicates by analysis of variance (ANOVA) using the general linear model (SAS 9.3, SAS Institute, Cary, NC). Least significant differences (LSD) were used to compare treatment mean values when significant differences (p<0.05) were found.

## Competing interests

The authors declare that they have no competing interests.

## Authors’ contributions

JB and SCL designed the project. JB performed the adventitious root and nanoparticle production and wrote the manuscript with SCL. SCL performed the imaging and characterization of the nanoparticles. CNS and MZ contributed to the overall project development and manuscript preparation. All authors read and approved the manuscript.
